# The allosteric gating mechanism of the MthK channel

**DOI:** 10.1093/nsr/nwac072

**Published:** 2022-04-13

**Authors:** Fenghui Guan, Tianyu Li, Wei Dong, Rui Guo, Hao Chai, Zhiqiu Chen, Zhong Ren, Yang Li, Sheng Ye

**Affiliations:** Frontiers Science Center for Synthetic Biology (Ministry of Education), Tianjin Key Laboratory of Function and Application of Biological Macromolecular Structures, School of Life Sciences, Tianjin University, Tianjin 300072, China; The Cancer Hospital of the University of Chinese Academy of Sciences, Institute of Basic Medicine and Cancer (IBMC), Chinese Academy of Sciences, Hangzhou 310022, China; State Key Laboratory of Drug Research and Key Laboratory of Receptor Research, Shanghai Institute of Materia Medica, Chinese Academy of Sciences, Shanghai 201203, China; University of Chinese Academy of Sciences, Beijing 100049, China; Life Sciences Institute, Zhejiang University, Hangzhou 310058, China; Department of Logistics, Tianjin University, Tianjin 300072, China; State Key Laboratory of Drug Research and Key Laboratory of Receptor Research, Shanghai Institute of Materia Medica, Chinese Academy of Sciences, Shanghai 201203, China; University of Chinese Academy of Sciences, Beijing 100049, China; University of Chinese Academy of Sciences, Beijing 100049, China; Department of Chemistry, University of Illinois at Chicago, Chicago, IL 60607, USA; Renz Research Inc., Westmont, IL 60559, USA; State Key Laboratory of Drug Research and Key Laboratory of Receptor Research, Shanghai Institute of Materia Medica, Chinese Academy of Sciences, Shanghai 201203, China; University of Chinese Academy of Sciences, Beijing 100049, China; National Clinical Research Center for Aging and Medicine, Huashan Hospital, Fudan University, Shanghai 200040, China; Frontiers Science Center for Synthetic Biology (Ministry of Education), Tianjin Key Laboratory of Function and Application of Biological Macromolecular Structures, School of Life Sciences, Tianjin University, Tianjin 300072, China; Life Sciences Institute, Zhejiang University, Hangzhou 310058, China

**Keywords:** ion channel, allosteric gating mechanism, protein structural data analysis

## Abstract

Allostery is a fundamental element during channel gating in response to an appropriate stimulus by which events occurring at one site are transmitted to distal sites to regulate activity. To address how binding of the first Ca^2+^ ion at one of the eight chemically identical subunits facilitates the other Ca^2+^-binding events in MthK, a Ca^2+^-gated K^+^ channel containing a conserved ligand-binding RCK domain, we analysed a large collection of MthK structures and performed the corresponding thermodynamic and electrophysiological measurements. These structural and functional studies led us to conclude that the conformations of the Ca^2+^-binding sites alternate between two quaternary states and exhibit significant differences in Ca^2+^ affinity. We further propose an allosteric model of the MthK-gating mechanism by which a cascade of structural events connect the initial Ca^2+^-binding to the final changes of the ring structure that open the ion-conduction pore. This mechanical model reveals the exquisite design that achieves the allosteric gating and could be of general relevance for the action of other ligand-gated ion channels containing the RCK domain.

## INTRODUCTION

K^+^ channels are multi-subunit allosteric membrane proteins that transit between closed and open states in response to an external stimulus in a process known as gating [1–5]. A remarkable feature of K^+^ channels is that they display a cooperative phenomenon during the gating process [6–9]. Such switch-like behavior is critical for the physiological functions of K^+^ channels, which permits a much more sensitive response to a small change in the external stimulus in the cellular context.

MthK is a Ca^2+^-gated K^+^ channel from *Methanobacterium thermoautotrophicum* that contains a conserved C-terminal ligand-binding domain termed the RCK domain for its role in regulating the conductance of K^+^ [10,11]. Previous electrophysiological studies of MthK gating have demonstrated a steep Ca^2+^ dependence, consistent with a positive inter-subunit cooperativity and strong energetic coupling between Ca^2+^-binding and channel opening [12–14]. The unusually high Hill coefficient of the MthK channel suggests that multiple Ca^2+^-binding events are strongly coupled. Such a cooperative mechanism drives the gating process of the channel in response to external stimuli.

**Figure 1.**
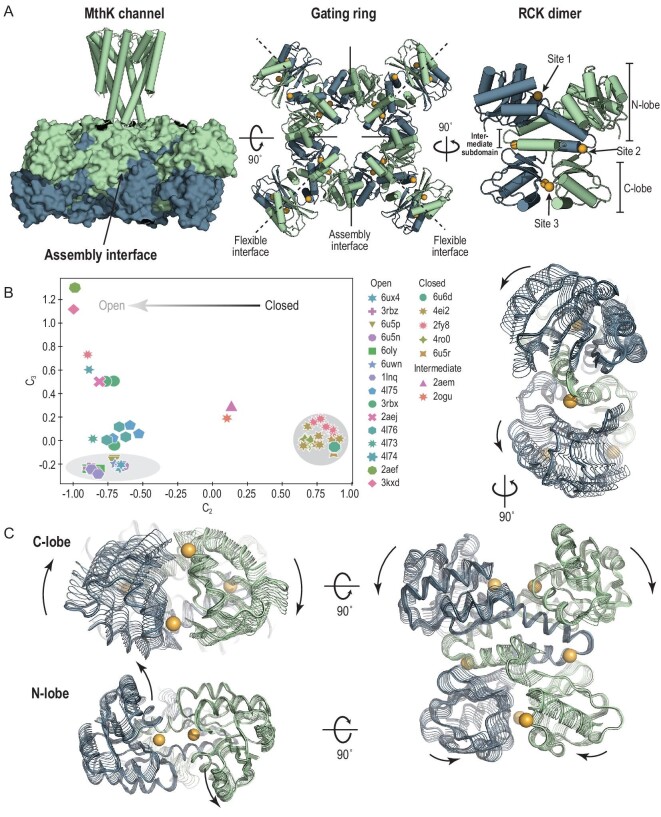
The dominant allosteric motion that RCK dimer undergoes during state transition. (A) A functional MthK channel requires eight RCK domains to form an octameric gating ring on the intracellular side of the pore (PDB ID: 6UX4). There are three Ca^2+^-binding sites in each RCK domain; thus, a gating ring of the MthK channel contains a total of 24 Ca^2+^-binding sites. (B) RCK-dimer structures projected onto the second and third modes. Each point (or structure) is colored according to its PDB ID. The open and closed RCK dimers mainly differ in composition of the second eigenvector, C_2_. Two RCK dimers (PDB ID: 2OGU, 2AEM) are interpreted as intermediate during the allosteric transition. The ring-forming RCK dimers are highlighted by gray shadowing. (C) Visualization of the second eigenvector characterizing the dominant motion that the RCK dimer undergoes during closed-to-open state transition.

The gating ring of the MthK channel is the central apparatus responsible for the cooperative gating. A functional MthK channel requires eight RCK domains to form an octameric gating ring on the intracellular side of the tetrameric ion channel pore (Fig. 1A). The eight RCK domains belong to two different groups: the top four in green are linked to the pore-forming peptide chains, whereas the bottom four in blue are co-expressed from the MthK gene using an internal starting site (Met107) and are co-assembled in the cytosol. Two RCK domains, one from each group, form an RCK dimer through an extensive interface, termed the flexible interface [15]. Four RCK dimers assemble a gating ring through the assembly interfaces between neighboring dimers. The flexible and assembly interfaces alternate around the ring and hold the eight RCK domains in an enclosed ring architecture (Fig. 1A). The gating ring undergoes large quaternary changes between the Ca^2+^-free closed and the Ca^2+^-bound open states, with a change in the gating-ring diameter of >8 Å [16]. The expansion of the gating ring from closed to open exerts a lateral force on the pore-lining inner helices leading to the opening of the channel. This large structural change originates from the interaction of Ca^2+^ ions with protein at local sites involving only a few residues. One Ca^2+^-binding site (primary site, or Site 1) was initially identified in each RCK domain located at the base of the cleft between two RCK domains [15] and subsequently two additional ones (Sites 2 and 3) were revealed at the peripheral subdomain protruding out from the gating ring (Fig. 1A) [17]. Thus, a gating ring of the MthK channel contains a total of 24 Ca^2+^-binding sites, corresponding to the unusually high Hill coefficient of the MthK channel.

How are ligand-induced structural changes that originate in one subunit transmitted to the others to structurally modulate their ligand-binding affinities? Though the structures of stable conformational states of the MthK-gating ring are known [14,15,18–20], the mechanism by which ligand-induced structural changes propagate through the molecule remains elusive. The traditional approach based on pairwise structural comparisons had demonstrated that the gating ring undergoes large quaternary changes between different states. To deduce a quantitative description of allostery, we carried out a joint analysis of all available structures, aiming to get a big picture of ‘the whole elephant’ rather than biased perspectives of specific parts of the subject with limited scopes [21]. This analysis, which is equivalent to a principal component analysis, revealed an allosteric gating mechanism of MthK that was further verified by thermodynamic and electrophysiological experiments.

## RESULTS

### The joint analysis reveals the dominant allosteric motion of the RCK dimer during state transition

The MthK-gating rings in two discrete states, the Ca^2+^-free closed and the Ca^2+^-bound open, have been constantly observed in both crystal and cryo-EM structures [14,15,18–20]. As the basic unit of the gating ring, the RCK dimer also exhibits discrete states [22–25]. We collected 88 RCK-dimer conformers from 22 PDB entries (Supplementary Tables 2 and 3) determined in this study (Supplementary Table 1) and previously. They could be divided into two groups (Supplementary Figure 1)—one group that consists of RCK dimers in closed gating rings is referred as ‘closed’ and the other one as ‘open’. Conformational differences have been observed within and between groups, although the crucial changes underlying the ligand-induced closed-to-open allosteric transition of the RCK dimer remain obscure. Thus, we used a well-established statistical technique, the singular value decomposition (SVD), to capture the essential features in the structure data set [21,26]. We first built a structure data matrix A of 88 columns, with each column representing a conformer structure in the form of interatomic distances between all Cα-atoms along the protein backbone. We then applied SVD to the data matrix, while the resulting orthogonal eigenvectors, or in other words principal components, describe the axes of maximal variance of the distribution of structures. Projection of the distribution onto the subspace defined by a handful of eigenvectors with the largest eigenvalues results in a lower dimensional representation of the structural data set. These low-dimensional representations, here termed ‘conformer plots’, succinctly display the relationships between different conformers, highlight the major differences between structures and enable the interpretation and characterization of multiple inter-conformer relations (Supplementary Figure 2).

As shown in Fig. 1B, the closed and open groups are separated along the second dimension, suggesting that they mainly differ in composition of the second eigenvector, u_2_. Thus, u_2_ bears the conformational features that distinguish the closed and open states, describing the closed-to-open motion of the RCK dimer (Supplementary Figure 3). This dominant motion is illustrated in Fig. 1C, mainly composed of the rotations of the conserved N-terminal Rossman fold domain (N-lobe) and the less conserved C-terminal domain (C-lobe), around the axes across the linking helix-turn-helix domain (intermediate domain). The most interesting aspect revealed by the conformer plot is an intermediate state in between closed and open states. Compared with typical closed and open conformers, the arrangement of two N-lobes in the intermediate-state RCK dimers resembles that of open conformers, while the arrangement of dimeric C-lobes is opposite (Supplementary Figure 4). This suggested that the rotations of the N-lobe and the C-lobe within a subunit could be decoupled during allosteric transition, whereas the two N-lobes, as well as the two C-lobes, always move collectively.

### The specific bilobed architecture amplifies the subtle side-chain displacements to the significant quaternary structural differences

**Figure 2.**
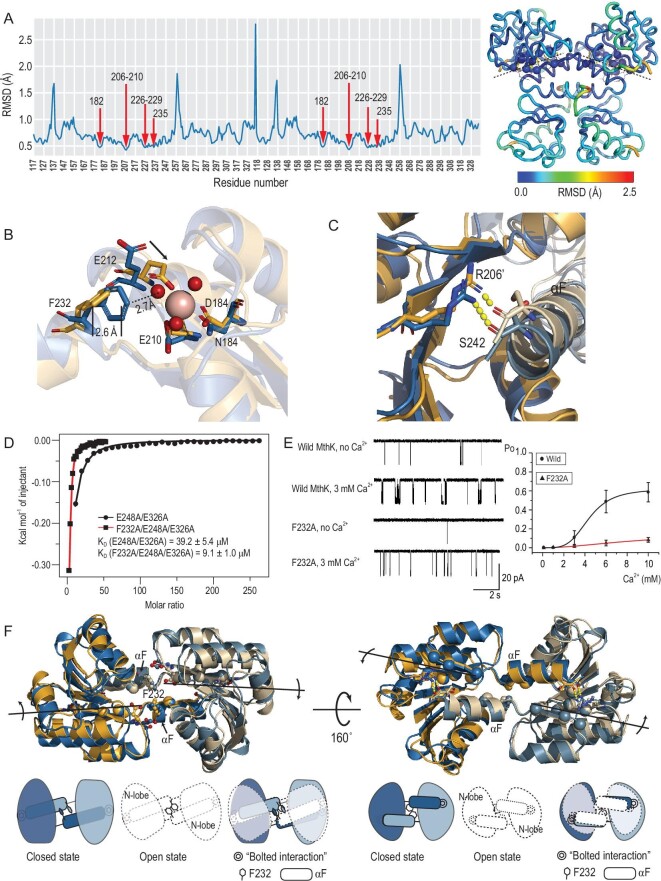
The specific bilobed architecture transmits and amplifies the Ca^2+^-binding-induced conformational changes. (A) The mobility profile of the RCK dimer. Left: the average cumulative RMSD of each residue is plotted. Right: the RCK-dimer structure is colored according to each residue's average RMSD, with immobile residues (R206-E210, D226, Q227, I229, I182, S235) shown as spheres. Hydrogen bonds between immobile residues are shown as yellow dashed lines, whereas the black dashed lines represent the rotation axes of N-lobes. (B) Alternative stereochemical Ca^2+^-binding modes between closed (blue color, PDB ID: 2FY8) and open (yellow color, PDB ID: 2AEF) states at Site 1. The bound Ca^2+^ ion (wheat) and Ca^2+^-chelating water molecules (red) are shown as spheres. At Site 1, the swinging-back of E212 and the relative displacement of F232 upon Ca^2+^ binding are indicated. (C) Helix αF is tightly associated with the neighboring N-lobe by hydrophobic interactions and the ‘bolted interaction’, a hydrogen bond between R206 and S242. (D) ITC experiments to measure the Ca^2+^-binding affinities of the MthK-gating ring mutants. The total heat exchanged during each injection is fitted to a single-site binding isotherm with K_D_ as an independent parameter, where K_D_ = 39.2 μM for E248A/E326A and K_D_ = 9.1 μM for F232A/E248A/E326A. Similar values for K_D_ are obtained using different protein concentrations varied over a 5-fold range. (E) Left: single-channel traces of wild-type and F232A MthK channels with or without the addition of 3 mM internal [Ca^2+^]. Membrane voltage was –100 mV so that channel openings cause a downward current. Right: plots of channel open probability (Po) as a function of [Ca^2+^] for MthK WT and F232A mutant, recorded at pH 7.5. The smooth lines fit with the Hill equation, *Po* = *P*_max_/(1 + (K_1/2_/[Ca^2+^])*^n^*), where *n* is the Hill coefficient and K_1/2_ is the [Ca^2+^] required for Po to reach half of maximum. For WT MthK, *n* = 4 and K_1/2_ = 4.4 mM; for F232A MthK, *n* = 1.8 and K_1/2_ = 9.5 mM. (F) Two N-lobes with extending helices αFs form a specific bilobed architecture that is central for allosteric transition. Ca^2+^ binding at one primary site triggers the side-chain displacements of F232 and E212, generating a repulsive force that rotates the N-lobe around the axis across β strand βE and the helix αF movement. Since the N-lobe is tightly associated with the neighboring helix αF, the conformational changes that originate in one subunit are transmitted to the other subunit, which facilitates the second Ca^2+^-ion binding. Bottom: schematic drawing illustrates the allosteric coupling between two N-lobes and helices αFs. The closed state is colored light blue and dark blue for two subunits, while the open state is shown as dashed white graphics. The RCK dimer is shown in closed (left), open (middle) and overlap (right) states.

We profiled the mobility of all residues and determined the rotation axis of the N-lobe. By calculating the root mean square deviation (RMSD) of every Cα–Cα pair distances, we generated a RMSD map in which the average of each column represents the residue's mobility (Fig. 2A). The relatively immobile residues, with respect to the minimum average RMSD, mainly distributed at two adjacent β strands in the N-lobe, which defines the rotation axis. The identification of the rotation axis and its proximity to the primary Ca^2+^-binding site (Site 1) became key to understanding how the small structural changes triggered by Ca^2+^-binding are amplified to the significant conformational changes of the MthK-gating ring.

To figure out the original ligand-binding-induced displacements, we compared RCK-dimer structures in different states and observed two alternative stereochemical Ca^2+^-binding modes at Site 1 that may exhibit significant differences in Ca^2+^-binding affinity. Superimposition of immobile residues revealed two substantial differences (Fig. 2B). First, the side chain of Glu212 chelating the Ca^2+^ ion in the open state swings away in the closed state. Second, the side chain of Phe232 is ∼1.7 Å closer to the Ca^2+^-binding site in the closed state than that in the open state. The chelation of a Ca^2+^ ion at Site 1 involves three acidic residues (Asp184, Glu210 and Glu212) and three water molecules, coordinated with an apparently bipyramidal geometry [22]. These allow us to reasonably imagine a Ca^2+^-binding process at Site 1: a Ca^2+^ ion in its hydrated form first enters; three water molecules in the inner hydration shell of the Ca^2+^ ion are then displaced by the carboxylate oxygens of Asp184 and Glu210, resulting in partial Ca^2+^ binding at Site 1; the side chain of Glu212 swings back to displace one more water molecule to achelate the Ca^2+^ ion. However, in the closed state, the aromatic ring of Phe232 is in close proximity to a Ca^2+^-chelating water molecule (∼2.7 Å) and the partially charged carboxylate oxygen of Glu212 (∼3.2 Å), which increases the energetic cost of Ca^2+^ binding at Site 1 in the closed state, by repelling the entering of a hydrated Ca^2+^ ion and the swinging-back of the side chain of Glu212. This characteristic suggests that the Ca^2+^ binding at Site 1 in the closed state is an energetically unstable configuration, likely associated with lower Ca^2+^-binding affinity, whereas in the open state, the ∼2.6-Å displacement of the aromatic ring of Phe232 leaves the Ca^2+^-binding at Site 1 energetically stable.

We substituted F232 for alanine on the RCK mutant (E248A/E326A) containing only Site 1 and carried out isothermal titration calorimetry (ITC) experiments to measure the Ca^2+^-binding affinities of the MthK-gating ring mutants. The heat transfer associated with each injection is plotted as a function of the ligand (Ca^2+^)-to-protein concentration ratio and fitted to an equation that incorporates the enthalpy and affinity of a single Ca^2+^-ion-binding event (see ‘Materials and methods’). For the RCK mutant (E248A/E326A) containing only Site 1, the fit corresponds to dissociation constant K_D_ = 39.2 μM. Ca^2+^ activation of the MthK channel requires mM-range Ca^2+^ concentration [12,13,15,17], while the Ca^2+^-binding affinity of the MthK-gating ring is in the μM range. The difference indicated that the ion-conduction pore exerts a constraint on the gating ring in the closed state, requiring more Ca^2+^ to open the channel. Importantly, the F232A mutant (F232A/E248A/E326A) displays a 4.3-fold higher Ca^2+^-binding affinity (K_D_ = 9.1 μM) than that with F232 (E248A/E326A) (Fig. 2D), confirming the hindrance effect of F232 on Ca^2+^ binding. However, abrogating the hindrance effect did not facilitate channel activation. As shown in Fig. 2E, the F232A single mutation leads to MthK channels with dramatically decreased Ca^2+^ sensitivity and Hill coefficients (*n* = 1.83 ± 0.11) compared with that of a wild-type channel (*n* = 4.39 ± 0.06), indicating a reduction in cooperativity. This proves the essential role of the aromatic ring of Phe232 in amplifying the small structural changes triggered by Ca^2+^ binding to the significant conformational changes of the MthK-gating ring.

In conclusion, Ca^2+^ binding at one of the two Ca^2+^-binding sites (Site 1) of an RCK dimer causes the side chain of Glu212 to swing back chelating the Ca^2+^ ion and becoming too close to the benzene ring of Phe232. The close proximity of a negatively charged carboxylate oxygen atom and a hydrophobic carbon atom effectively generates a repulsive force in between and expels the aromatic ring of Phe232 from the pocket. Two aromatic rings of Phe232s from both RCK subunits locate in the deep cleft between two N-lobes and are surrounded by hydrophilic residues, with limited space to undergo Ca^2+^-dependent side-chain rearrangement. To release the repulsive force, the only solution is to undergo a motion around the rotation axis.

The specific bilobed architecture of the RCK dimer ensures the collective motion of two N-lobes. A key feature of the RCK dimer is that two subunits are tightly associated and helix αF crosses over interlocking with the N-lobe of the neighboring subunit. Besides, two helices αFs are arranged in a staggered fashion without strong short-range interactions around Phe232 at the N-terminus, while they are connected to the neighboring N-lobe with a hydrogen bond between Arg206 and Ser242 (or Ile243) at the C-terminus (Fig. 2C). This inter-subunit interaction presents in all RCK dimers, so we referred to it as the ‘bolted interaction’. As shown in Fig. 2F, the bolted interactions and the flexible dimer hinge form a bilobed architecture. Because of this specific structure, each of the N-lobes moves along with the helix αF of the neighboring subunit and vice versa, consequently changing the angle between two αFs. The distribution of these angles (Supplementary Figure 5) shows a jump between the closed and open states, suggesting that dimeric N-lobes function as a toggle switch that transits between two discrete states. Furthermore, this means the first Ca^2+^ binding at Site 1 promotes the second Ca^2+^ binding at Site 1 within the dimer, meaning that two Ca^2+^ ions at Site 1 cooperatively stabilize the open conformation. This cooperative model could explain the decreased Ca^2+^ sensitivity and Hill coefficient of the F232A mutant MthK channel. And it also explains why E212Q mutation yields MthK channels with increased Ca^2+^ sensitivity, completely opposite to the effects of other charge-neutralizing mutations at Site 1 [17,25]. The amide group of Gln has larger van der Waals sphere than that of the carboxylate oxygen of Glu, thereby enhancing the repulsive force (Supplementary Figure 6) that facilitates the allosteric motion.

### Intra-dimer cooperativity

**Figure 3.**
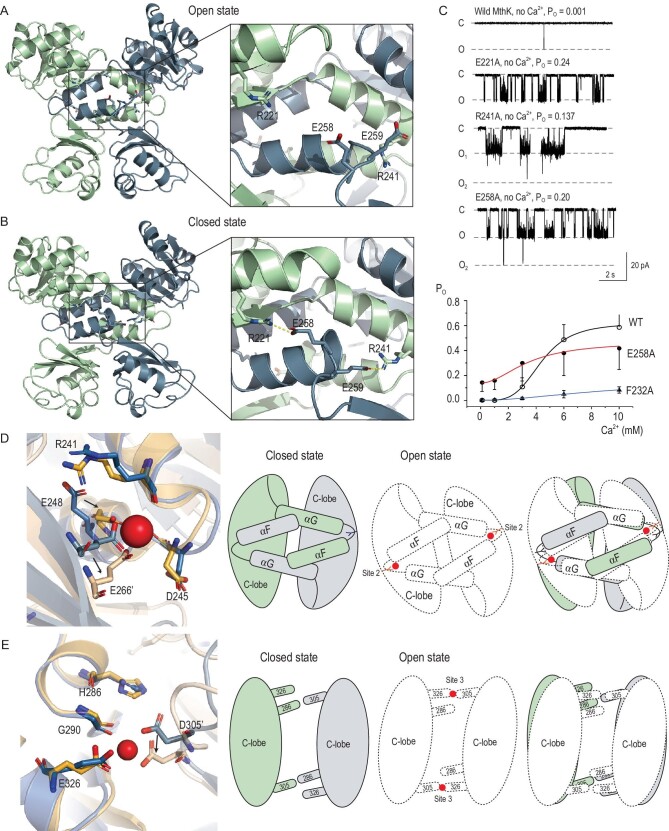
Intra-dimer cooperativity. (A) Open-state RCK dimer (PDB ID: 1LNQ) showing the disruption of the ionic lock (details in the inset). (B) Closed-state RCK dimer (PDB ID: 2FY8) showing intra-dimer ionic lock (details in the inset) that stabilizes the dimer in the closed state. (C) Single-channel traces of wild-type and mutant MthK channels without the addition of internal [Ca^2+^]. Membrane voltage was –100 mV so that channel openings cause a downward current. (D) and (E) Left: alternative stereochemical Ca^2+^-binding modes between closed (yellow color, PDB ID: 2FY8) and open (blue color, PDB ID: 3RBZ) at Site 2 (D) and Site 3 (E). Right: schematic drawings illustrating the allosteric coupling between different Ca^2+^-binding sites in an RCK dimer. The closed state is colored light blue and dark blue for two subunits, while the open state is shown as dashed white graphics. The RCK dimer is shown in closed (left), open (middle) and overlap (right) states. The C-lobes are shown as ovals, with helices αFs and αGs as rectangles. Two N-lobes are removed for clarity. (D) E266 is shown, while Ca^2+^ ions appear as red spheres, respectively. The rotation of helices αFs and N-lobes arising from Ca^2+^ binding at Site 1 directly transmits to helices αGs sliding and further to C-lobe twisting. These motions reposition E266 and other residues that are required for the formation of Ca^2+^ binding at Site 2. (E) Two N-lobes and helices αFs and αGs are removed for clarity. From closed to open states, the C-lobe twisting breaks the D305–H286’ hydrogen bond between subunits, causing the formation of the Ca^2+^-binding Site 3.

In addition to the primary Ca^2+^-binding site (Site 1) that we described before, there are two other Ca^2+^-binding sites in one RCK domain. Ca^2+^-binding Site 2 locates between the intermediate subdomain and the C-terminal lobe of the other subunit [17]. We observed that they switch ‘off’ in the closed state and ‘on’ in the open state. In the open state, at Site 2, the Ca^2+^ ion is chelated by the carbonyl oxygens of Arg241 and Asp245, two carboxylate oxygens of Glu248 from one subunit and two carboxylate oxygens of Glu266 from the neighboring subunit. While in the closed state, Glu266 has an ∼3-Å main-chain shift, which pushes the side chain of Glu248 swinging away, resulting a complete disruption of the stereochemistry at Site 2 (Fig. 3D). Similarly, at Site 3, the Ca^2+^ ion is chelated by the carbonyl oxygen of Gly290, two carboxylate oxygens of Glu326 from one subunit and two carboxylate oxygens of Asp305 from the neighboring subunit [17]. The stereochemistry is also disrupted in the closed state with Asp305 moving away by ∼2.7 Å (Fig. 3E). The two C-lobes twist around each other during closed-to-open state transition, Asp305 from each subunit has an ∼3-Å main-chain shift, breaking the hydrogen bond (Asp305–His286) between subunits, apparently causing the formation the Ca^2+^-binding Site 3 (Fig. 3E). Twisting of the C-lobe relative to the intermediate subdomain from the other subunit also causes a main-chain shift of Glu266 and the side chain of Glu248 swings back, forming the Ca^2+^-binding Site 2 (Fig. 3D).

The formation of two other Ca^2+^-binding sites relies on the rotation motion of C-lobes, which is likely coupled to the N-lobe rotation triggered by the Ca^2+^ binding at the primary sites, explaining the high cooperativity of the MthK channel. Then where is the hinge region that couples the N-lobe and C-lobe motions? Both u_2_ and the RMSD map reveal that the loop ^257^Ala-Glu-Glu-Ser^260^ between the intermediate subdomain and C-lobe undergoes dramatic rearrangement. This is confirmed by a crease-finding algorithm in which each RCK-dimer structure is aligned to the reference structure by least-squares fitting of five residues and the r.m.s.d. value is plotted as a function of the central residue number (Supplementary Figure 7) [27]. All of these results indicated that this link region is an intra-subunit hinge. Since the helix-turn-helix intermediate subdomains interact with each other and form a hydrophobic dimeric interface (the flexible interface), this loop is close to the neighboring subunit's N-lobe and helix αF, indicating that it may function as a diverter that propagates the structural perturbations across the flexible interface. By comparing RCK-dimer conformations, we find that in the open state, the backbone hydrogen bonds between Ala257 and Val252, Gln253 and Glu258 are disrupted, releasing these two residues from the helix αG and leading the hinge-loop swings away from the neighboring N-lobe. While in the closed state, Aal257 and Glu258 extend the helix αG and the loop interacts with the N-lobe of the neighboring subunit. Specifically, Glu258 forms a salt bridge (2.69 ± 0.23 Å) with Arg221 located at the neighboring N-lobe and Glu259 interacts with Arg241 (2.90 ± 0.25 Å) at the neighboring intermediate domain (Fig. 3B).

 

 

These interactions are disrupted in the open state (Fig. 3A). Thus, we named these interactions an ‘intra-dimer ionic lock’ for their role in maintaining the RCK dimer in its closed state. It seems that the concerted motion of N-lobes and αFs could break the ‘intra-dimer ionic lock’ and trigger the rearrangements of ^257^Ala-Glu-Glu-Ser^260^ loops, driving two αGs to slide toward each other and the C-lobes to rotate. We designed mutants that disrupted these interactions and observed higher open probabilities of the mutant channels without application of Ca^2+^ compared with that of  WT (Fig. 3C). This suggested that disruption of these ‘intra-dimer ionic locks’ could shift the equilibrium of the RCK dimer to the open state, which increases the intrinsic open probability of a mutant channel. However, the sensitivity to Ca^2+^ and the maximum open probability of the E258A mutant channel dramatically decreased (Fig. 3C). This suggested that the disturbed hinge region would affect the collective motion of the N-lobe and C-lobe, resulting in a reduction in cooperativity with a lower Hill coefficient (*n* = 1.97 ± 0.22).

### Inter-dimer cooperativity and the complete allosteric gating mechanism

**Figure 4.**
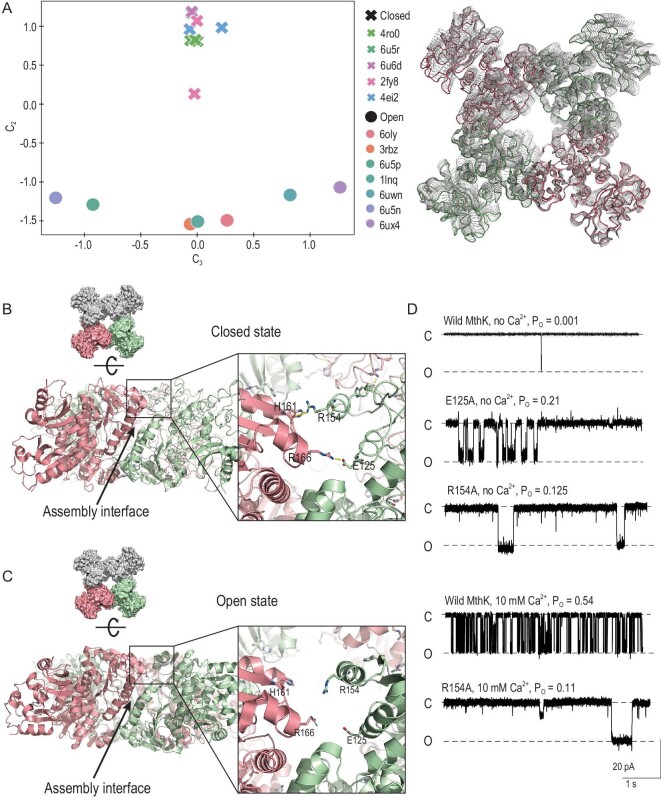
Inter-dimer cooperativity. (A) Left: the conformer plot of gating-ring structures. All structures are projected onto the second and third modes. The open and closed gating rings are separated along the second dimension, with a previously determined partially open gating ring (PDB ID: 2FY8) in between. The open rings are widely distributed along the third dimension, while the closed rings are clustered around zero. Right: conformational ensemble obtained from interpolating along the third modes. All structures, with adjacent RCK dimers colored differently, are superimposed with one subunit. This suggests that open-state gating rings display higher dynamics than closed-state gating rings with respect to the inter-dimer arrangement. (B) and (C) Side views of the closed state (PDB ID: 2FY8) showing the inter-dimer ionic lock (details in the inset) that stabilizes the assembly interface in the closed state (B) and the open-state gating ring (PDB ID: 1LNQ) showing the disruption of the ionic lock (details in the inset) (C). (D) Single-channel traces of wild-type and mutant MthK channels with or without the addition of internal [Ca^2+^]. Membrane voltage was –100 mV so that channel openings cause a downward current.

A functional MthK-gating ring is an octamer. To address how Ca^2+^ binding triggered the transition of one RCK dimer shifts the equilibrium between the closed and open states of the entire gating ring, we analysed all octameric ring structures by applying the same approach and found that all of them, except for a previously determined partially open ring [18], fall into either open-state or closed-state groups. Similarly to RCK dimers, the second dimension distinguished the closed and open ring structures. Surprisingly, it showed that along the third dimension, which mainly characterizes the distance differences between adjacent RCK dimers (Fig. 4A), the open octamers are widely distributed, whereas the closed octamers are tightly clustered (Fig. 4A). This suggested that, compared to the closed-state, the open-state gating ring displays greater variability in terms of the RCK-dimer arrangements.

We found that the neighboring RCK dimers in most closed gating rings form a short salt bridge between E125 and R166 (2.8 ± 0.2 Å) and a short hydrogen bond between the guanidinium moiety of R154 and the carbonyl oxygen atoms of H161 (3.2 ± 0.6 Å) (Fig. 4B), which are all disrupted in the open state (Fig. 4C). To test the effects of these interactions, we designed mutants that disrupt these interactions. The electrophysiological recordings showed that the open probabilities of mutant channels in the absence of Ca^2+^ dramatically increase compared with that of  WT (Fig. 4D), indicating that these interactions constrain the quaternary structure of the gating ring in the closed state. We therefore named these interactions an ‘inter-dimer ionic lock’. However, this ‘lock’ is absent in two closed-ring structures determined by cryo-EM recently, where the distance between the E125 and R166 is ∼4 Å [20]. Considering that, in these structures, the second transmembrane helices TM2s are straight and form a bundle that crosses at the intracellular side, it is reasonable to predict that these helices explore steric constraints as well as the ‘inter-dimer ion lock’.

The gating ring is stabilized in the closed state in the absence of Ca^2+^. As in a dimer, Ca^2+^ binding causes the rotational motions of two N-lobes in a dimer. Such rotation motions could drive the motion of neighboring N-lobes, breaking the inter-dimer ionic lock and exerting a lateral force that tugs the C-linker. This would remove the constraints holding the gating ring in the closed state and tip the equilibrium between the two alternative quaternary structures some way in favor of the open state. Consequently, the structural transition at the Ca^2+^-binding Site 1 in one subunit is directly transmitted to the neighboring dimer. Thus, the rearrangement of the assembly interface provides a pathway for communication between dimers, enabling the cooperative binding of Ca^2+^. This is partially supported by our electrophysiological recordings that the open probability of the R154A mutant channel in the presence of a high concentration of Ca^2+^ does not increase as the WT channel does (Fig. 4D). The outer rim of the gating ring expanded by ∼8 Å from closed to open states, resulting in an opening of the channel gate to ∼12 Å, large enough to allow hydrated K^+^ ions to pass freely [16]. This large conformational change would tug at the C-linker between the gating ring and the pore domain, allowing the TM2 helices to kink open at the hinge glycine. The channel is then opened by the associated unfolding of the last two helical turns of TM2 and the C-linker detaching from the RCK domain and becoming disordered [20].

## DISCUSSION

Despite fundamental importance in nearly every biological process, the structural mechanisms of protein functions have been difficult to elucidate. The basic problem has been the difficulty in inferring the dynamic structural motions of proteins. Here we presented a statistical approach to analyse all available snapshots of the dynamic process captured by structural biology. Pairwise structural comparison is susceptible to random structural variations due to the source of organisms, mutants, crystal forms and many other experimental conditions. In contrast, this ‘big-data’ analysis could isolate the common trend of structural motions relevant to function among profuse structural variations.

We proposed a model of cooperative activation in an MthK-gating ring that the two N-lobes and two αFs form a canonical hinged-dimer architecture that could transmit the ligand binding at one site to a concerted motion of two lobes that, in turn, increases the other site's affinity. An MthK-gating ring is composed of RCK domains that are particularly prevalent among prokaryotic ligand-gated K^+^ channels and major K^+^-selective transport systems [15,28–32]. Besides, RCK domains also exist in eukaryotic channels of the Slo gene family, which includes the high-conductance Ca^2+^-activated K^+^ channels (BK) [33,34], Na^+^-activated K^+^ channels (KCNT1-2) [35] and H^+^-inhibited K^+^ channel (KCNMC1) [36]. The wide distribution of RCK domains highlights their importance in regulating the flow of K^+^ across the cell membrane. A remarkable feature of K^+^ channels containing RCK domains is that they display a cooperative phenomenon during the gating process. Interestingly, all RCK domains share a similar overall architecture albeit with diverse ligands. Thus, the cooperative model that we concluded is likely to be a common mechanism of the RCK regulatory apparatus. KefC, the regulatory subunit of glutathione-gated K^+^ efflux transporter KefF, exhibits the same dimer-hinge conformational change [32]. The N-terminus of the helix is also connected to the neighboring subunit's Rossman fold subdomain, forming two ‘bolt’ regions with the hinge region in between. In addition, a similar architecture and conformational change are also observed in the cytosolic regulatory subunit of the Ktr-ion transporter [28].

In conclusion, this cooperative model not only provides a structural basis that explains the cooperative behavior of the MthK channel, but also suggests a general mechanism for transmitting motions and signals across an interface between subunits and domains. Nevertheless, the combination of structural analyses with functional verifications presented here may be a valuable tool for addressing functional motions in a wide variety of macromolecular systems.

## MATERIALS AND METHODS

### Protein purification

*MthK* gating ring was over-expressed and purified as previously described [16].

### Crystallization and structure determination

The purified protein was concentrated to ∼6 mg/mL for crystallization. Crystals were grown using sitting-drop vapor diffusion at 20ºC by mixing equal volumes of protein and reservoir solution of 0.4 M KCl, 0.1 M Tris, pH 8.5 and 20%–25% PEGMME 350. Crystals were cryo-protected from their mother liquid by increasing the concentration of PEGMME 350 to 40% and were frozen in liquid nitrogen. All diffraction data were collected at the Shanghai Synchrotron Radiation Facility (SSRF) BL17U beamline (Shanghai, China). The data were indexed, integrated and scaled using the program HKL-2000 [37]. Phases were determined by molecular replacement using PHASER [38] with the closed state gating ring (PDB ID: 2FY8) as a search model. Model adjustment was done iteratively using COOT [39], and structure refinement was done using REFMAC [40].

### Joint analysis of RCK structures on a large scale

All PDB entities are downloaded as biological assemblies; sequences of all chains are aligned by ClustalO. Based on the multiple sequence alignment, the distances between every two Cα atoms are calculated. The lower triangles of the distance matrices are assembled into columns of matrix ***A***. SVD factors matrix ***A*** into three component matrices, where the factorization has the form ***UΣV^T^***. The *i*th column in ***A***, ${a_i} \approx \mathop \sum \nolimits_{j\ = \ 1}^k {v_{ij}}{s_j}{{\boldsymbol{u}}_{\rm{j}}}$, where ${v_{ij}}$ is the (*i, j*) entry of the matrix ***V***, ${s_j}$ is the singular value in ***Σ*** and ${{\boldsymbol{u}}_{\rm{j}}}$ is the *j*th column in ***U***. Thus, we define ${v_{ij}}{s_j}$ as the coefficient ${c_j}$, representing the projection on the *j*th left singular vector ${{\boldsymbol{u}}_{\rm{j}}}$, which is visualized by conformer plot.

### ITC measurement and fitting

Measurements of the heat exchange associated with Ca^2+^ binding to both MthK-gating ring mutants were acquired using a microcalorimeter (VP-ITC; GE Healthcare). All experiments were performed at a constant temperature of 25°C. The sample cell was filled with protein solutions including 250 mM NaCl, 20 mM Tris, pH 8.0, whereas the injector contained the same buffer with 2–5 mM CaCl_2_. The data were fitted to a one-site binding model in the Origin program. The affinities were reported as K_D_ (or 1/K) in the text and figures.

### Electrophysiological studies

The purified MthK channel and its mutants were reconstituted into lipid vesicles composed of POPE and POPG at a protein-to-lipid ratio of 1–2 μg/mg using the same method as described [41]. The detergent was slowly removed by dialysing for 48 hours. A vertical lipid bilayer set-up was used to record the activities of MthK channels as previously described. Membrane voltages were clamped and currents were recorded using an Axopatch 200B amplifier with a Digidata 1322A analog-to-digital converter (Axon Instruments). Current was sampled at 10 kHz and low-pass filtered at 2 kHz. Software TAC (Bruxton) was used in statistical analysis of single-channel data.

## Supplementary Material

nwac072_Supplemental_Files
