# Supplementary material for: The allosteric gating mechanism of the MthK channel
Source: Natl Sci Rev. 2022 Apr 13;9(8):nwac072. doi: 10.1093/nsr/nwac072 (PMC9440719; doi:10.1093/nsr/nwac072)
Supplement: nwac072_Supplemental_Files [file nwac072_supplemental_files.docx]

**Supplemental data for “Novel insights into the allosteric gating mechanism of MthK channel”**

**Materials and Methods**

**Protein expression and purification**

We obtained the soluble RCK domain from the soluble fraction of *E.coli* cell lysates in which the *MthK* gene was overexpressed, by taking advantage of the fact that the *MthK* gene expressed in *E. coli* also produces the soluble RCK domain in excess to the channel. MthK gene was cloned into a pQE70 vector (Qiagen) with a thrombin cleavage site between the channel and the C-terminal hexahistidine tag. The channel was overexpressed in *E.coli* SG13009 cultures by induction with 0.4 mM isopropyl-β-D-thiogalactopyranoside (IPTG) at A_600_~0.8 and cells were harvested and lysed in 50 mM Tris^.^HCl, pH 8.0, 250 mM NaCl containing Leupeptin, Pepstatin, Aprotinin, and PMSF (Sigma) to inhibit proteases. The supernatant of the cell lysate containing an excess of the RCK domain was collected after centrifugation at 21,000g for 20 minutes. The RCK domain was purified on a Talon Co^2+^ affinity column (Clontech) and eluted with 20 mM Tris^.^HCl, pH 8.0, 250 mM NaCl, and 300 mM imidazole. Protein eluted from the Co^2+^ column was incubated for three hours at room temperature (~21 ˚C) in the presence of 1.0 unit of thrombin (Roche) per 2.0 mg of protein to remove the hexahistidine tag, and further purified on a superdex 200 (10/30) size-exclusion column (Pharmacia) in 20 mM Tris^.^HCl, pH 8.0, 250 mM NaCl.

**Crystallization and structure determination.**

The purified protein was concentrated to about 6 mg/ml using a 10 kDa cutoff Amicon Ultra (Millipore) for crystallization. Crystals were grown by sitting-drop vapor diffusion at 20 ºC by mixing equal volumes of protein and reservoir solution of 0.4 M KCl, 0.1 M Tris, pH 8.5 and 20-25% PEGMME 350. Crystals were cryo-protected from their mother liquid by increasing the concentration of PEGMME 350 to 40%, and were frozen in liquid nitrogen. All diffraction data were collected at the Shanghai Synchrotron Radiation Facility (SSRF) BL17U beamline (Shanghai, China). The data were indexed, integrated, and scaled using the program HKL-2000 [37].

The crystals are in space group P2_1_ and contain fourty subunits forming five octameric gating ring per asymmetric unit. The best crystal diffracted X-rays to 3.2 Å resolution, with unit-cell dimensions of a=166.3 Å, b=232.7 Å, c=198.0 Å, β=94.6º. Phases were determined by molecular replacement using PHASER [38] with the closed state gating ring (PDB ID: 2FY8) as a search model. Model adjustment was done iteratively using COOT [39], and structure refinement was done using REFMAC [40]. The presence of multiple subunits in the asymmetric unit of the crystals provides a significant enhancement of the accuracy of the crystallographic analysis: first, by enabling averaging of the electron density over multiple crystallographically independent regions of the molecular replacement map, and second, by providing a powerful set of constraints on the atomic model during refinement. The models were refined with data to 3.2 Å resolution, maintaining highly restrained stereochemistry and keeping tight non-crystallographic symmetry restraints. The final models contain forty RCK molecules. All structural illustrations were prepared with PYMOL ([www.pymol.org](http://www.pymol.org)).

**Joint analysis of RCK structures on a large scale.**

Although protein crystallography has been successfully applied to structural study for decades, a major shortcoming of this technology is its intrinsic limiting for static state and stringent lactic periodicity. Crystal structures provide snapshots of molecules in static states, hence missing the information of dynamic structure motions. Therefore, to elucidate the progression of cooperative structure motions and allosteric regulation of MthK octameric gating ring, we applied a joint-analysis approach based on a large collection of RCK structures including various states, conditions and space groups [7, 21, 26, 27]. All PDB entries are downloaded as biological assemblies, sequences of all chains are aligned by ClustalO. The RCK dimers and gating rings were identified manually, each of them is denoted as a conformer. Based on the alignment, the distances between every two Cα atoms are calculated from the Cartesian coordinates. The lower triangles of the distance matrices are assembled into columns of matrix ***A***. SVD factors an M$\times$N matrix ***A*** into three component matrices, where the factorization has the form ***UΣV^T^***. The column in ***U***, $\boldsymbol{u}_{j}$, is the i^th^ left singular vector that bears the conformational features distinguish the conformers. The approximation of ***A*** can be reconstructed by the first k columns in ***U*** and ***V*** together with the corresponded singular values in ***Σ***. In other words, the i^th^ column in ***A***, $a_{i}\approx\sum_{j=1}^{k} v_{ij}s_{j}\boldsymbol{u}_{j}$, where $v_{ij}$ is the (*i, j*) entry of the matrix ***V***, $s_{j}$ is the singular value in ***Σ***, and $\boldsymbol{u}_{j}$ is the j^th^ column in ***U***. Thus, we define $v_{ij}s_{j}$ as the coefficient $c_{j}$, representing the projection on the j^th^ left singular vector $\boldsymbol{u}_{j}$, which is visualized by conformer plot.

**ITC measurement and fitting**

Measurements of the heat exchange associated with Ca^2+^-binding to both MthK gating ring mutants were acquired using a microcalorimeter (VP-ITC; GE Healthcare). All experiments were performed at a constant temperature of 25°C. All solutions were filtered and degassed before each experiment. The sample cell (V=1.4301 ml) was filled with protein solutions including 250 mM NaCl, 20 mM Tris, pH 8.0, whereas the injector containing the same buffer with 2-5 mM CaCl_2_, 25–30 injections were performed with 5 µl of ligand injected into sample cell each time. The data were fit to a one-site binding model in the Origin program. A constant background was subtracted. The affinities were reported as K_D_ (or 1/K) in the text and figures.

**Electrophysiological studies**

The purified MthK channel and its mutants were reconstituted into lipid vesicles composed of 1-palmitoyl-2-oleoyl-phosphatidylethanolamine (POPE, 7.5 mg/ml) and 1-palmitoyl-2-oleoyl-phosphatidylglycerol (POPG, 2.5 mg/ml) at a protein-to-lipid ratio of 1-2 µg/mg using the same method as described [41]. The dialysis buffer (300mM KCl, 4mM NMG and 10mM HEPESs, pH 7.0) was used to slowly remove the detergent from the detergent/lipid/protein mixture in 48 hours. A vertical lipid bilayer setup was used to record the activities of MthK channels as previous described. The paint lipids consisted of POPE and POPG (in a ratio of 3:1) were solved in decane and then were painted over a hole (~150 μm) in a polystyrene partition separating the internal and external solutions. The channel activities were recorded under symmetrical 150 mM KCl and 10 mM HEPES, pH 7.5 on both side of solutions. Without additional application of [Ca^2+^] or EGTA, and the free [Ca^2+^] concentration in solution would be about 10mM. Membrane voltages were clamped and currents were recorded using an Axopatch 200B amplifier with a Digidata 1322A analogue-to-digital converter (Axon Instruments). Current was sampled at 10 kHz and low-pass filtered at 2 kHz. Software TAC (Bruxton) was used in statistical analysis of single channel data.

**
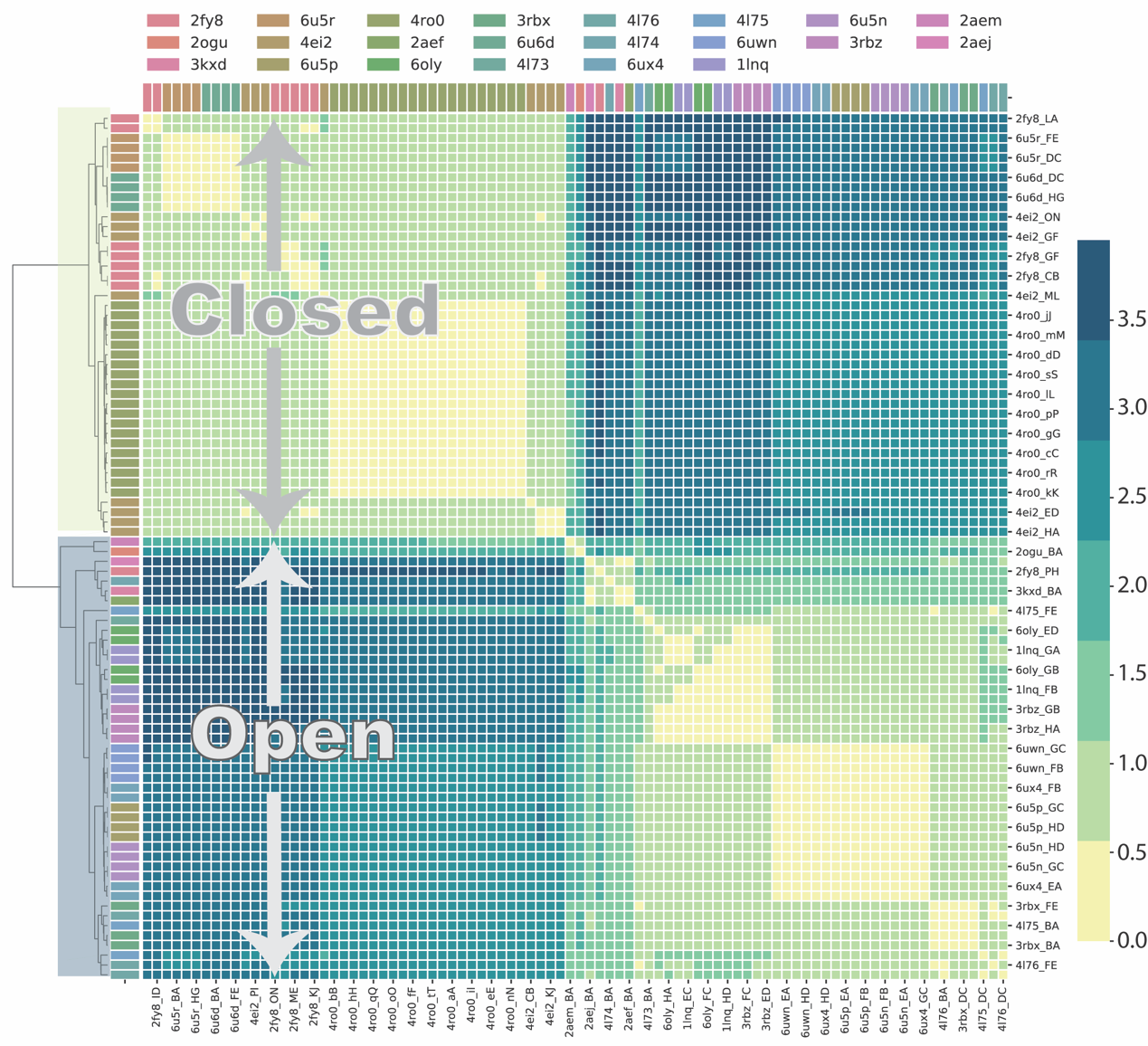
**

**Figure 1. Heatmap of structure differences between RCK dimers.** A primary means of measuring structure differences is alignment of molecule structures by least-squares fitting. We use the superimpose module in biopython to align C_α_ trace of one RCK dimer onto another and calculate the RMSD value. The RMSD for each pair of structures is indicated with a linear color gradient as shown in the color bar on right of the map. Annotations on left of the heatmap show the result of hierarchical clustering, demonstrating all RCK dimers can be divided into two groups.

**
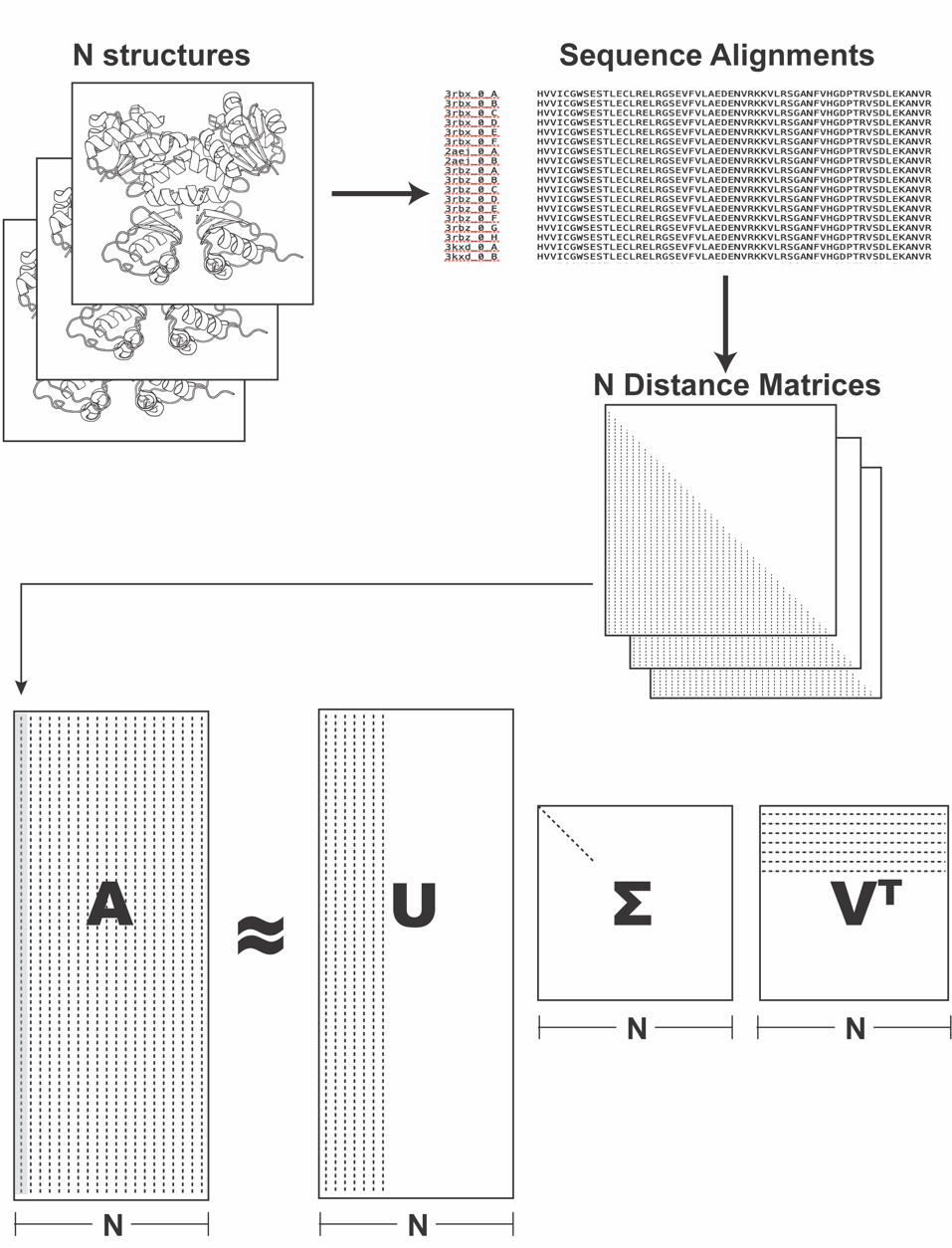
**

**Figure 2. A workflow of the joint-analysis.** All PDB entries are downloaded as biological assemblies, sequences of all chains are then extracted and aligned. Based on the alignment, the distances between every two Cα atoms are calculated from the Cartesian coordinates. The lower triangles of the distance matrices are assembled into columns of matrix ***A***. SVD factors an M$\times$N matrix ***A*** into three component matrices, where the factorization has the form ***UΣV^T^***. The approximation of ***A*** can be reconstructed by the first k columns in ***U*** and ***V*** together with the corresponded singular values in ***Σ***. In other words, the i^th^ column in ***A***, $a_{i}\approx\sum_{j=1}^{k} v_{ij}s_{j}\boldsymbol{u}_{j}$, where $v_{ij}$ is the (*i, j*) entry of the matrix ***V***, $s_{j}$ is the singular value in ***Σ***, and $\boldsymbol{u}_{j}$ is the j^th^ column in ***U***. Thus, we define $v_{ij}s_{j}$ as the coefficient $c_{j}$, representing the projection on the j^th^ left singular vector $\boldsymbol{u}_{j}$.

**
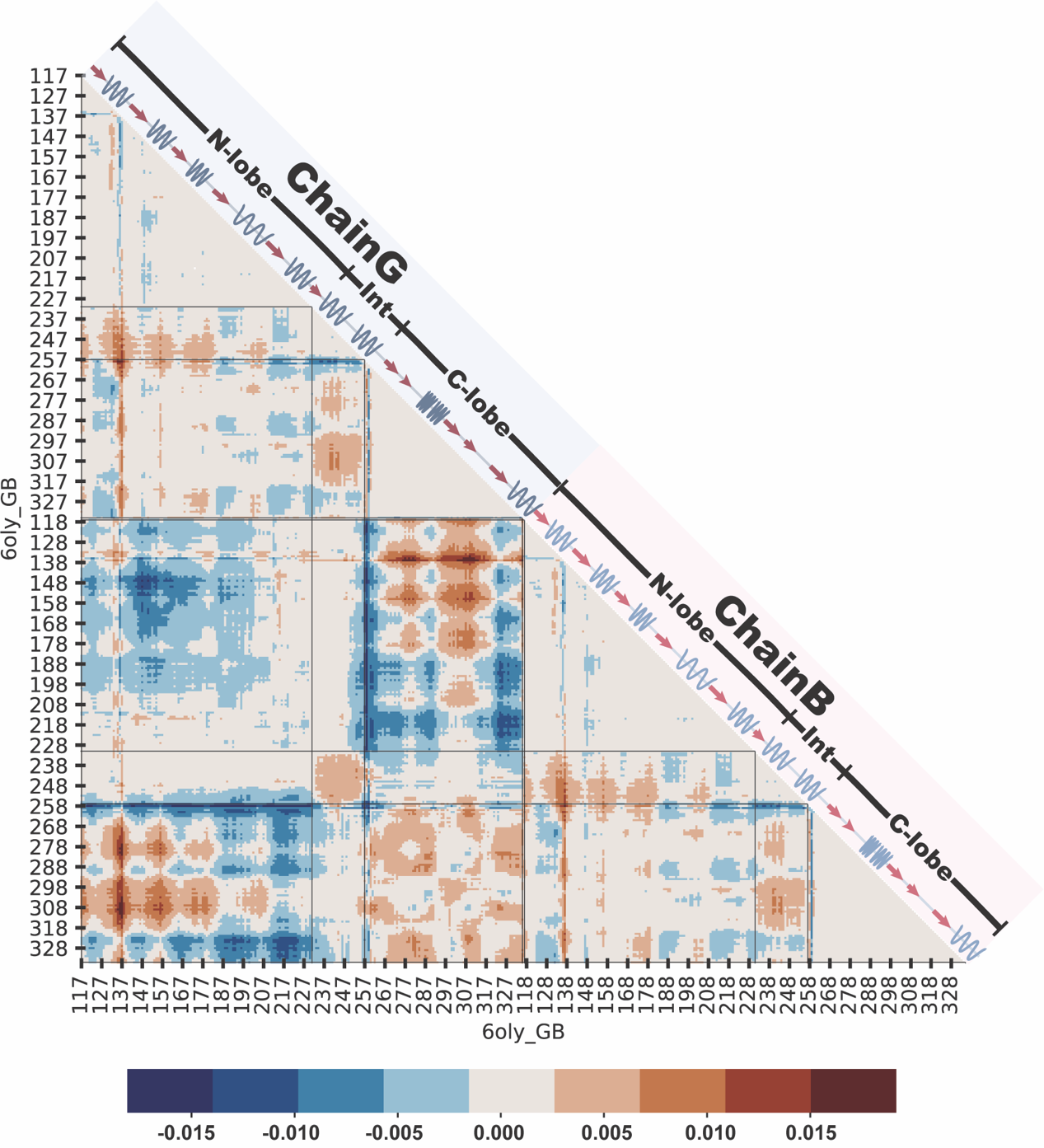
**

**Figure 3. The second left eigenvector U_2_ of RCK dimer structural dataset.** The second left singular vector bears the conformational features that distinguish the open and closed state. The N-lobes and C-lobes undergo orientational changes dramatically, while with insignificantly positional changes.


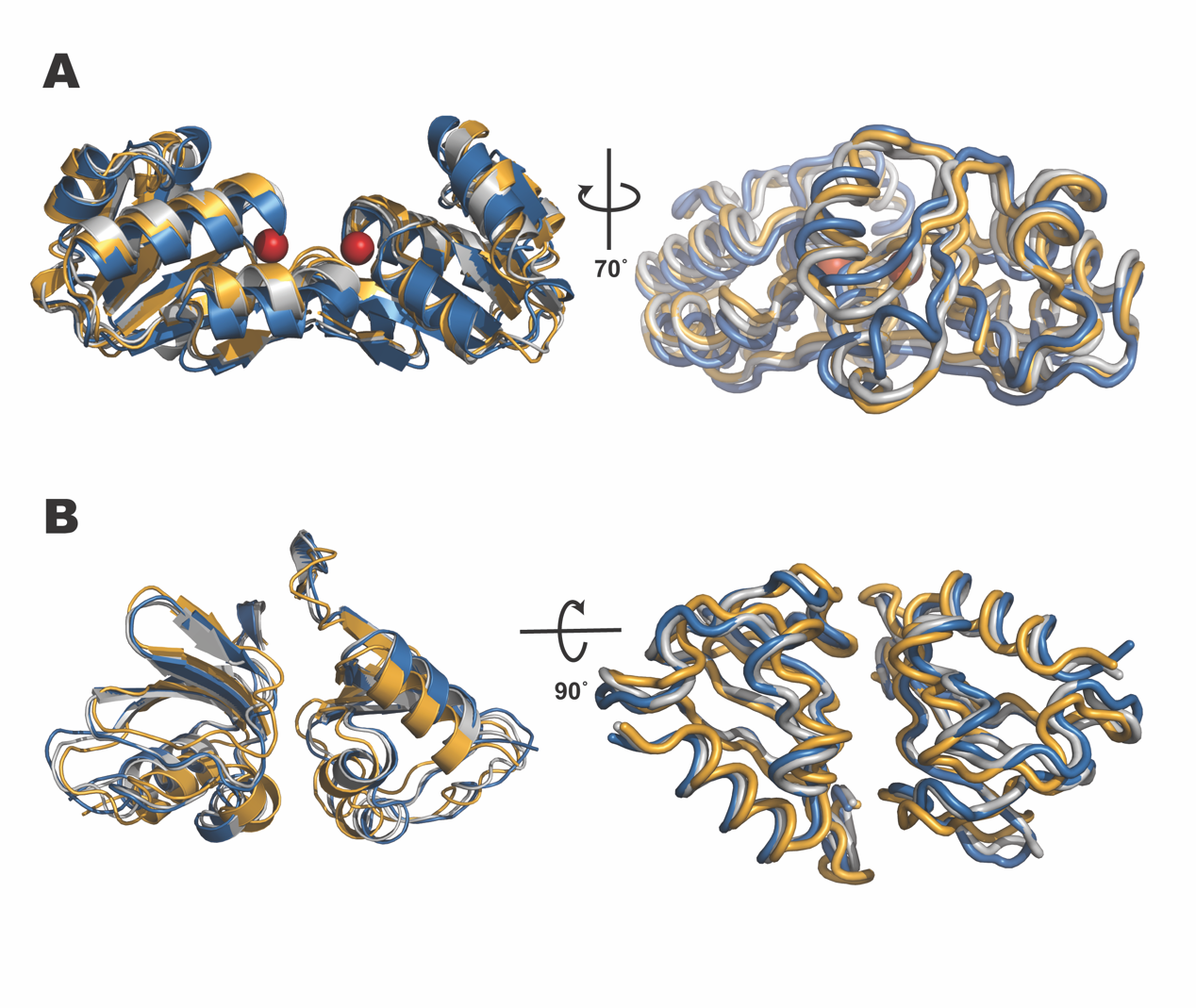


**Figure 4. The N-lobes conformation of intermediate state resembles the open state while the C-lobes conformation is opposite.** The open state RCK dimer (PDB ID: 1LNQ, colored yellow) is superimposed on the intermediate state RCK dimer (PDB ID: 2AEM, colored white), as well as the closed state RCK dimer (PDB ID: 2FY8, colored blue). **(A)** The RMSD between the N-lobes of intermediate and closed state is 1.90Å, and that between the intermediate and open state is 1.00Å. **(B)** The RMSD between the C-lobes of intermediate and closed state is 0.82Å, and that between the intermediate and open state is 2.07Å.


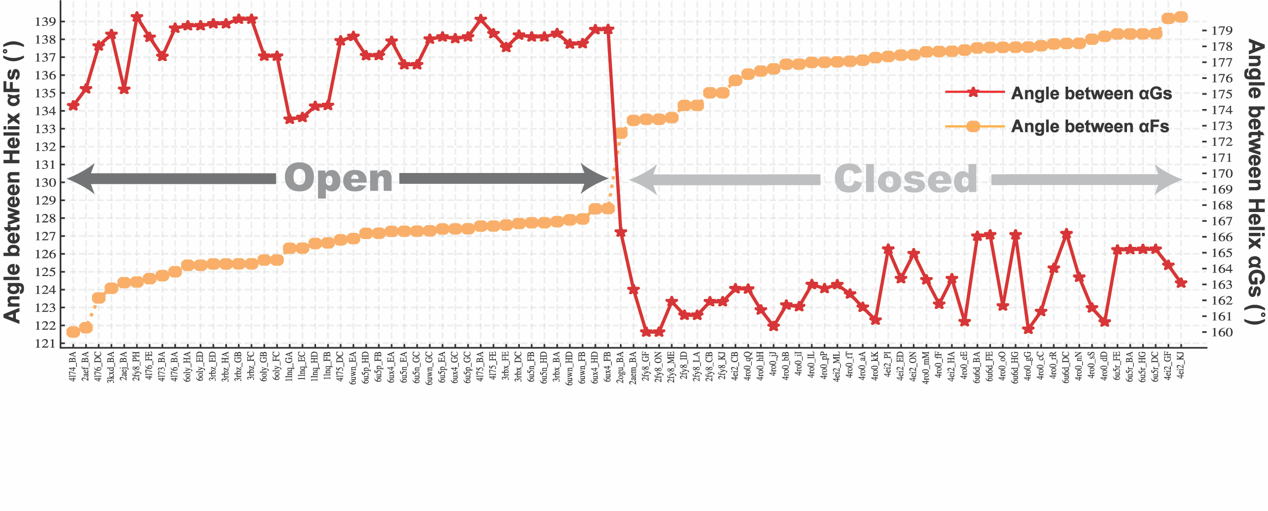


**Figure 5. Allosteric coupling of the N-lobe rotation and the intermediate subdomain helices sliding.** The helix axis is calculated following the method of Sugeta and Miyazawa (H. Sugeta and T. Miyazawa, Biopolymers 5, 673-679 (1967).). The angle between two helices is given by acos of the dot product of the two direction vectors, and is plotted as function of the PDB ID. The discrete distribution of angles between two αFs and αGs demonstrates that the assembly interface alternates between two discrete states, closed and open.


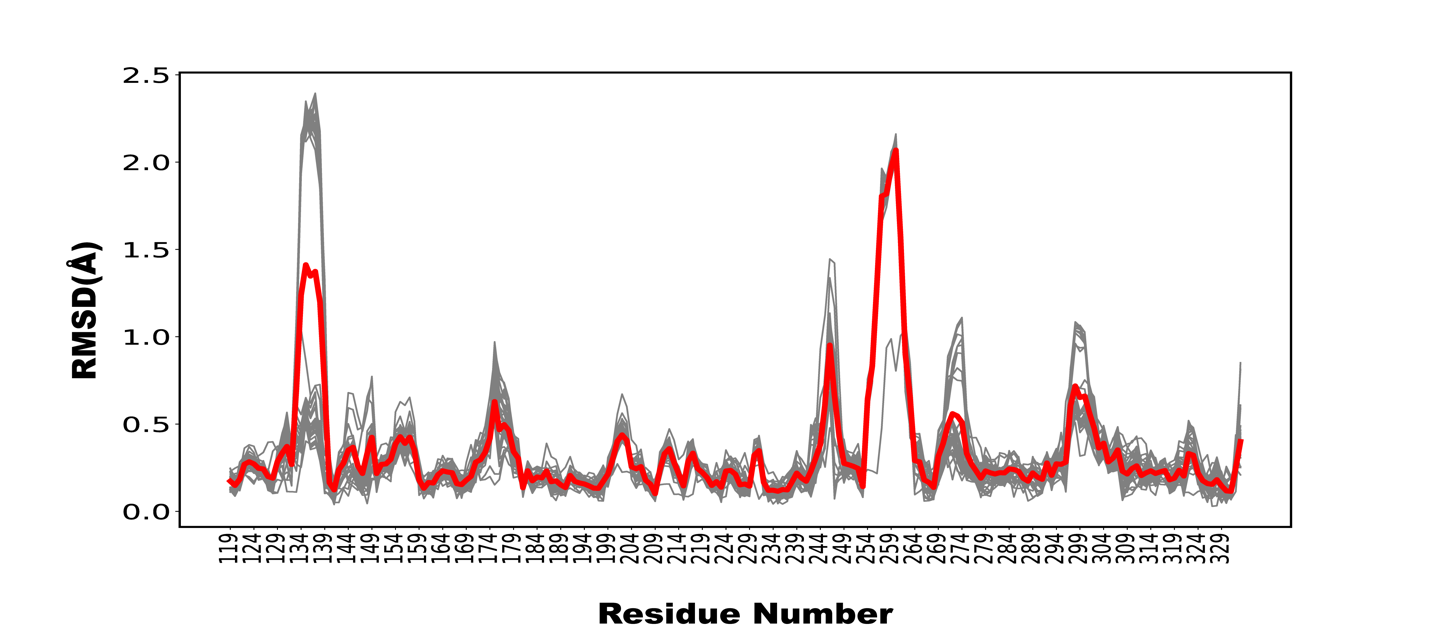


**Figure 6. The hinge region identified by a crease-finding algorithm.** Each open state RCK dimer structure is aligned to a reference structure (2FY8_BA) by least-squares fitting of from -2 to $+$2 residues around a given residue. The rmsd value of the fitting is plotted as function of the central residue ID. A representative fitting using the open state RCK structure (1LNA_GA) is colored in red, and all other fittings in gray. The short segments involved in least-squares fitting make the curve sensitive to local crease while insensitive to rigid body motion. Large rmsd value indicates hinge region of a local segment.


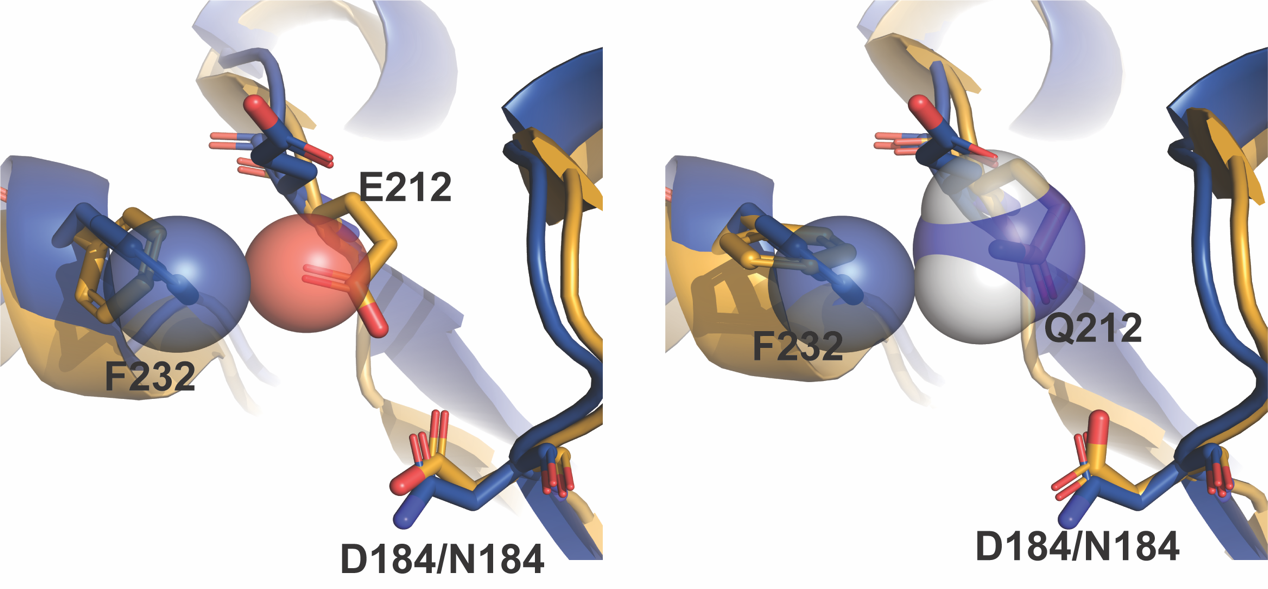


**Figure 7. The enhanced repulsion effect of Q212.** Left, superposition of closed (blue, PBD ID: 2FY8) and open state (yellow, PDB ID: 1LNQ) RCK domains showing the repulsion between F232 and the swinging back E212 upon Ca^2+^-binding. The Cζ atom of Phe232 and the carboxylate oxygen atom of Glu212 side chain are shown as spheres in CPK representation, highlighting an obvious clash in between. Right, superposition of closed (blue, PDB ID: 2FY8) and open state (yellow, PDB ID: 4L76) RCK domains showing the enhanced repulsion between F232 and swinging back Q212 upon Ca^2+^-binding. The Cζ atom of Phe232 and the side chain amide group of Q212 are shown as spheres in CPK representation.

**Table 1. X-ray data and refinement statistics**

| Data collection | |
| --- | --- |
| Space group  Unit cell  Resolution (Å)  Measured reflections  Unique reflections  Redundancy  Completeness (%, highest shell)  Mean I/σI (highest shell)  Rsym (%, highest shell) | P2_1_  a=166.3 Å,  b=231.7 Å  c=198.0 Å  β=94.6°  3.2  918,851  229,804  4.0  92.8 (93.4)  10.8 (1.8)  16.1 (74.2) |
| Refinement | |
| Resolution (Å)  No. of reflections \|F\|>0 σF  R-factor/R-free (%)  No. of atoms  Protein  Water  Average B-factors (Å^2^)  r.m.s. deviations  Bond lengths (Å)  Bond angles (°)  Ramachandran plot  Most favored regions (%)  Additional allowed regions (%)  Generously allowed regions (%)  Disallowed regions (%) | 3.2  227378  26.3/30.1  68748  0  79.7  0.006  1.103  90.8  9.2  0  0 |

**Table 2. Available MthK gating ring structures**

| PDB ID | Protein | Gating ring/AU, Conformational states | ligand | Mutation | Resolution (Å) | Reference |
| --- | --- | --- | --- | --- | --- | --- |
| 1LNQ | MthK | 1, open | Ca^2+^ | M107I | 3.3 | [1] |
| 2FY8 | MthK RCK | 2, One closed, the other one partially open | n/a | D184N | 2.8 | [2] |
| 3RBZ | MthK | 1, open | Ca^2+^ | M107I | 3.4 | [3] |
| 4EI2 | MthK RCK | 2, closed | Ba^2+^ | - | 3.1 | [4] |
| 6OLY | MthK | 1, open | Ca^2+^ | M107I | 3.1 | [5] |
| 6U5R | MthK | 1, closed | Ca^2+^ | - | 3.6 | [6] |
| 6U6D | MthK | 1, closed | n/a | - | 3.6 | [6] |
| 6UWN | MthK RCK | 1, open | Ca^2+^ | - | 3.5 | [6] |
| 6U5N | MthK RCK | 1, open | Ca^2+^ | - | 3.2 | [6] |
| 6UX4 | MthK RCK | 1, open | Ca^2+^ | - | 3.5 | [6] |
| 6U5P | MthK RCK | 1, open | Ca^2+^ | - | 3.3 | [6] |
| 4RO0 | MthK RCK | 5, closed | n/a | - | 3.2 | This study |

**Table 3. Available MthK RCK dimer structures**

| PDB ID | Protein | RCK dimer/AU, Conformational state | ligand | Mutation | Resolution (Å) | Reference |
| --- | --- | --- | --- | --- | --- | --- |
| 2AEF | MthK RCK | 1, open | Ca^2+^ | - | 1.7 | [7] |
| 2AEJ | MthK RCK | 1, open | n/a | - | 2.1 | [7] |
| 2AEM | MthK RCK | 1, intermediate | n/a | - | 2.8 | [7] |
| 3KXD | MthK RCK | 1, open | Cd^2+^ | - | 2.2 | [8] |
| 3RBX | MthK RCK | 3, open | Ca^2+^ | D184N | 2.8 | [3] |
| 4L73 | MthK RCK | 1, open | Ca^2+^ | - | 2.5 | [9] |
| 4L74 | MthK RCK | 1, open | Ca^2+^ | - | 1.8 | [9] |
| 4L75 | MthK RCK | 3, open | Ca^2+^ | D184N | 2.4 | [9] |
| 4L76 | MthK RCK | 3, open | Ca^2+^ | E212Q | 3.0 | [9] |
| 2OGU | MthK RCK | 1, intermediate | n/a | - | 3.2 | [10] |

**References**

1. Jiang, Y. *et al.* Crystal structure and mechanism of a calcium-gated potassium channel. *Nature* **417**, 515–22 (2002).

2. Ye, S., Li, Y., Chen, L. & Jiang, Y. Crystal structures of a ligand-free MthK gating ring: insights into the ligand gating mechanism of K+ channels. *Cell* **126**, 1161–73 (2006).

3. Pau, V. P. *et al.* Structure and function of multiple Ca2+-binding sites in a K+ channel regulator of K+ conductance (RCK) domain. *Proc Nat Acad Sci U S A* **108**, 17684–17689 (2011).

4. Smith, F. J., Pau, V. P., Cingolani, G. & Rothberg, B. S. Crystal structure of a Ba(2+)-bound gating ring reveals elementary steps in RCK domain activation. *Structure* **20**, 2038–47 (2012).

5. Kopec, W., Rothberg, B. S. & Groot, B. L. de. Molecular mechanism of a potassium channel gating through activation gate-selectivity filter coupling. *Nat Commun* **10**, 5366 (2019).

6. Fan, C. *et al.* Ball-and-chain inactivation in a calcium-gated potassium channel. *Nature* **580**, 1–6 (2020).

7. Dong, J., Shi, N., Berke, I., Chen, L. & Jiang, Y. Structures of the MthK RCK domain and the effect of Ca2+ on gating ring stability. *J Biol Chem* **280**, 41716–24 (2005).

8. Dvir, H., Valera, E. & Choe, S. Structure of the MthK RCK in complex with cadmium. *J Struct Biol* **171**, 231–7 (2010).

9. Smith, F. J., Pau, V. P., Cingolani, G. & Rothberg, B. S. Structural basis of allosteric interactions among Ca2+-binding sites in a K+ channel RCK domain. *Nat Commun* **4**, 2621 (2013).

10. Kuo, M. M., Baker, K. A., Wong, L. & Choe, S. Dynamic oligomeric conversions of the cytoplasmic RCK domains mediate MthK potassium channel activity. *Proc Nat Acad Sci U S A* **104**, 2151–6 (2007).
